# Personalised Blood Glucose Time Series Forecasting in Type 1 Diabetes: Deep Collaborative Adversarial Learning

**DOI:** 10.3390/jpm16040210

**Published:** 2026-04-08

**Authors:** Heydar Khadem, Hoda Nemat, Jackie Elliott, Mohammed Benaissa

**Affiliations:** 1 Department of Electronic and Electrical Engineering, University of Sheffield, Sheffield S5 7AU, UKm.benaissa@sheffield.ac.uk (M.B.); 2 Department of Oncology and Metabolism, University of Sheffield, Sheffield S5 7AU, UK; 3 Sheffield Teaching Hospitals, Diabetes and Endocrine Centre, Northern General Hospital, Sheffield S5 7AT, UK

**Keywords:** personalised diabetes management, deep learning, time series forecasting, blood glucose prediction, artificial intelligence

## Abstract

**Background/Objectives:** Blood glucose prediction (BGP) for individuals with type 1 diabetes (T1D) is a clinically essential yet highly challenging task in time series forecasting (TSF) and an important problem in personalised medicine. Accurate bespoke BGP is crucial for individualised T1D management, reducing complications, and supporting patient-specific glycaemic risk mitigation. However, the pronounced volatility of glycaemic fluctuations in T1D, combined with the need for mathematical rigor and clinical relevance, hampers reliable prediction. This complexity underscores the demand to explore and enhance more advanced techniques. While adversarial learning is adept at modelling intricate data variability, its potential for BGP remains largely untapped. **Methods:** This work presents a novel approach for BGP by addressing a key limitation in conventional adversarial learning when applied to this task. Typically, these methods optimise prediction accuracy within a set horizon by minimising adversarial loss. This focus overlooks how predictions align with longer-term patterns, which are critical for clinical relevance in BGP, thereby yielding suboptimal results. To overcome this limitation, we introduce collaborative augmented adversarial learning, designed to improve the model’s temporal awareness. Incorporating collaborative interaction optimisation, this approach enables the model to reflect extended time dependencies beyond the immediate horizon, thereby improving both the clinical reliability of predictions and overall predictive performance. We develop and evaluate four learning systems for BGP: independent learning, adversarial learning, collaborative learning, and adversarial collaborative learning. The proposed systems were evaluated for two clinically relevant prediction horizons, namely 30 min and 60 min ahead. **Results:** The interdependent collaboratively augmented learning frameworks, validated using the well-established Ohio T1D datasets, demonstrate statistically significant superior performance in both clinical and mathematical evaluations. **Conclusions:** Beyond advancing BGP accuracy and clinical reliability, the proposed approach supports personalised medicine by improving subject-specific glucose forecasting from CGM data, with potential relevance for more individualised diabetes monitoring and decision support. The proposed approach also opens new avenues for advancements in other complex TSF domains, as outlined in our future work.

## 1. Introduction

Time series (TS) is a sequence of data points indexed by time, typically collected at consistent intervals [1]. TS analysis involves developing methodologies to extract meaningful insights and patterns from this temporal data [2,3]. A key area within TS analysis is time series forecasting (TSF), which focuses on projecting historical trends and patterns into future time stamps [4]. TSF has a wide range of applications across various fields, including healthcare [5]. This drives extensive research efforts aimed at establishing robust forecasting frameworks [6,7,8]. Numerous studies have successfully developed robust TSF models across diverse domains, incorporating classical techniques such as autoregressive models, moving averages, exponential smoothing, and autoregressive integrated moving average [9,10,11,12,13,14].

Traditional TSF approaches, while widely utilised, face several limitations, including challenges in tuning, a heavy reliance on domain expertise, restricted capacity to capture complex patterns, and limited ability to handle outliers and missing values [15,16]. As a result, there has been a shift toward more advanced algorithms, particularly machine learning (ML), to enhance TSF capabilities [17,18,19,20]. Various ML techniques, such as penalised linear methods and nonlinear regression trees, have been explored for developing more effective TSF models [21,22,23,24].

Deep learning (DL), a subset of ML, has proven to be a powerful tool for complex computational tasks [25,26,27,28,29,30]. Its capacity to model nonlinear dynamics has made DL particularly effective in TSF applications [31,32]. Furthermore, advancements in data collection technology have led to the accumulation of large time series datasets, a crucial resource for DL analysis [33,34]. Consequently, studies have employed DL architectures like recurrent neural networks (RNNs), convolutional neural networks (CNNs), and transformers to develop robust TSF models [35,36,37,38,39,40]. Despite the demonstrated success of DL-based TSF models, further refinement and improvement remain critical, driving the development of more advanced interdependent techniques with enhanced analytical potential beyond independent techniques.

One such advancement is the incorporation of adversarial learning (AL) [41]. Initially designed for generating image data, AL represents an intricate DL training framework consisting of two subnetworks: a generator and a discriminator. The generator aims to produce realistic data, while the discriminator distinguishes between real and synthetic data. These subnetworks engage in an adversarial loop, where the generator progressively improves its ability to produce convincing outputs, and the discriminator becomes more adept at distinguishing real from synthetic data [42].

Building on its success in image synthesis, AL has been adapted for time series data generation and forecasting [43,44,45,46]. In these adaptations, a main generator predicts future values based on historical data, while a discriminator evaluates the similarity between the predicted values and the actual data [47,48,49,50,51,52]. These innovations have established AL as a promising tool for improving TSF models [41].

Enhancing advanced techniques like AL is essential for addressing real-world challenges, such as predicting blood glucose (BG) levels in individuals with type 1 diabetes (T1D) [53]. This TSF task is particularly complex [54], and continuous improvements are critical [55] for effective diabetes management [56,57,58]. Accurate BG predictions (BGP) can help reduce both acute and chronic complications associated with T1D [59,60]. However, the volatility of BG values poses significant challenges, underscoring the need for more advanced models capable of providing accurate and reliable forecasting. At the same time, the widespread use of wearable devices, such as continuous glucose monitoring (CGM) systems, enables the automated collection of vast datasets necessary for training such advanced models [61]. This technological development creates an ideal environment for the application of advanced techniques like AL, making them increasingly valuable for BGP [62].

BGP is also closely connected to personalised medicine, because glucose dynamics, glycaemic variability, and treatment responses differ substantially across individuals with T1D. As a result, clinically useful forecasting systems should not only be accurate in general but should also support subject-specific prediction and individualised glycaemic risk management using patient-level CGM data.

In this context, this paper introduces an interdependent learning framework for blood glucose prediction that combines adversarial and collaborative optimisation. The study is designed to address two methodological questions: first, whether adversarial training alone improves sequence-to-sequence blood glucose prediction relative to independent learning; and second, whether augmenting adversarial training with a collaborative post-horizon objective improves the temporal relevance of predictions. To this end, we evaluate four frameworks—independent learning (IL), adversarial learning (AL), collaborative learning (CL), and adversarial collaborative learning (ACL)—under identical preprocessing, data partitioning, optimisation, and evaluation conditions. In addition to this controlled internal comparison, the best-performing proposed framework is also benchmarked against established approaches reported in the literature, including classical statistical, machine-learning, feed-forward neural network, and recurrent neural network baselines. This design allows the contribution of the proposed learning strategy to be assessed separately from differences in data handling or training conditions. To assess the framework under practically relevant forecasting conditions, experiments were conducted for two prediction horizons, 30 min and 60 min ahead. These horizons were selected because they represent short-term forecasting intervals that are highly relevant in diabetes management: 30 min supports near-term anticipatory decision-making, while 60 min provides a more challenging but clinically valuable longer warning window.

## 2. Adversarial Learning in Time Series

This section provides an overview of representative recent work on adversarial learning (AL) and deep learning for time-series analysis and forecasting. In addition to individual application studies, recent survey papers have highlighted the rapid expansion of deep learning for time-series forecasting, including recurrent, convolutional, and transformer-based models, as well as the growing role of generative adversarial approaches in modelling, forecasting, augmentation, and imputation. For a broader overview of these developments, several dedicated reviews are already available in the literature [41,63,64,65].

The article [66] presents a promising new approach for modelling financial TS data using AL. The article first argues that traditional models, such as autoregressive or moving average techniques, struggle to capture the complex dynamics of financial data. Then, in response, the work proposes using AL and shows that this approach outperforms traditional models in terms of accuracy and ability to capture complex patterns in the data. Additionally, the article explores potential applications of AL in various financial domains, including fraud detection.

Another article [67] proposes a novel DL methodology that employs AL for anomaly detection in TS data. The authors assess the efficacy of the method on benchmark datasets and demonstrate its superior performance over several state-of-the-art anomaly detection techniques. This research introduces a promising solution for detecting anomalies in TS data, with potential applications across various domains.

A different study proposes a new approach to addressing the problem of missing values in multivariate TS data [68]. The authors introduce a specialized AL architecture designed for imputing TS data. The evaluation analysis of real-world datasets demonstrates that the model outperforms several existing imputation methods. This research offers a promising solution to the challenge of imputing missing values in multivariate time series data that has practical applications in diverse fields, such as finance, healthcare, and environmental monitoring.

Another research article [69] introduces a method for predicting hourly photovoltaic power output using a conditional AL. The proposed approach addresses the issue of limited training data by using conditional AL to generate synthetic data, which helps to increase the size of the training dataset. A DL model then uses the augmented dataset to predict the future output. The study compares the performance of this approach with other popular TSF methods and finds that it outperforms them in terms of accuracy. The new method’s potential for improving the accuracy of photovoltaic power forecasting could play a crucial role in integrating renewable energy sources into the power grid.

Article [70] introduces a new approach for TS prediction and classification using a combination of AL and recurrent neural networks with an attention mechanism. The recurrent neural network unit is used to analyse the temporal patterns in the data, while the AL unit is employed to create synthetic data that can be used to enhance the training dataset. Comparative analysis with other prevalent methods for TS prediction and classification demonstrates the superior accuracy and efficiency of this novel approach.

Finally, ref. [43] presents a new method for TS resampling that addresses the issue of unevenly spaced data. The proposed method uses AL to generate synthetic data that can fill in the gaps between the original data points. AL is trained to learn the statistical patterns of the original data and then used to generate synthetic data points that complete the missing values. The study compares the performance of this method with other popular techniques used for resampling TS data, and the results show that it is more accurate. The authors conclude that this new approach has the potential to improve the accuracy of time series resampling.

More broadly, recent deep learning studies in time-series forecasting have shown that forecasting performance can benefit from increasingly expressive architectures, including recurrent, convolutional, hybrid, and transformer-based models. In parallel, review papers on GANs for time series have shown that adversarial approaches are being used not only for direct forecasting, but also for sequence generation, augmentation, imputation, anomaly detection, and representation learning. Within healthcare and glucose forecasting specifically, recent studies have explored recurrent and multitask forecasting models, as well as GAN-based modelling of CGM-related signals. These developments confirm both the momentum of deep-learning-based forecasting and the emerging relevance of adversarial techniques for biomedical time series.

In conclusion, AL has found application in certain TSF domains. However, their use in BGP, where reliable forecasting is vital for diabetes management, remains underexplored. Additionally, conventional adversarial learning often yields suboptimal results in BGP, focusing primarily on adversarial loss within a limited prediction horizon and frequently neglecting longer-term trends that are clinically critical.

It is important to distinguish the proposed approach from existing GAN-based sequence models and multi-step forecasting strategies. In standard adversarial TSF frameworks, the discriminator evaluates whether predicted sequences within the PH are distributionally consistent with real sequences. This adversarial objective improves realism but does not explicitly encourage predictions to be informative of future trends beyond the PH boundary. Multi-step forecasting approaches, on the other hand, extend the prediction range by directly forecasting additional future steps; however, they treat the extended horizon as an end in itself, optimising accuracy across the entire extended window simultaneously.

The proposed collaborative component operates on a fundamentally different principle. Rather than extending the PH or modifying the adversarial objective, it introduces an auxiliary regressor that consumes the primary regressor’s PH predictions and uses them to forecast post-PH values. Critically, during training, the collaborative loss is backpropagated through the auxiliary regressor to the primary regressor, compelling it to embed extended temporal information within its original PH outputs. The primary regressor’s PH length and output remain unchanged at inference; the improvement arises because the training process has shaped its predictions to be more temporally coherent. This indirect enrichment of PH predictions through post-PH supervision is, to our knowledge, a novel training strategy that has not been explored in adversarial TSF literature.

## 3. Learning Systems Architecture

In this research, we develop and evaluate four learning frameworks for blood glucose prediction (BGP): independent learning (IL), adversarial learning (AL), collaborative learning (CL), and adversarial collaborative learning (ACL). These frameworks are built on three core components: a primary regressor, an auxiliary discriminator, and an auxiliary regressor. The novelty of the proposed framework lies in the learning strategy rather than in introducing a new sequence backbone. Standard GAN-based forecasting typically couples a predictor or generator with a discriminator so that predicted sequences better match the distribution of the target horizon. By contrast, our framework adds an auxiliary regressor that receives the predicted horizon and is trained to forecast the subsequent post-horizon window. This creates a collaborative objective that regularises the primary regressor toward predictions that are not only accurate and distributionally plausible within the prediction horizon, but also informative for what happens immediately afterwards. For clarity, this differs from ordinary direct multi-step forecasting, in which a single model is trained to predict a longer horizon directly; here, the beyond-horizon requirement is imposed indirectly through an interacting auxiliary regressor.

The primary regressor is responsible for the main task of BGP—processing a specified length of historical data and predicting future data within a predefined PH. The sequence-to-sequence forecasting capability of the primary regressor independently allows for extended insights when paired with auxiliary components. In this study, a multilayer perceptron (MLP), well-known for its efficiency in BGP [71], is utilised as the primary regressor. This choice was deliberate. The aim of this paper is to evaluate the proposed learning framework rather than to maximise performance through increasingly complex temporal backbones. After reframing the CGM series into fixed history-to-horizon input-output pairs, the ordered lag values already encode short-term temporal structure, allowing a lightweight MLP to serve as a stable and interpretable baseline for testing the effect of adversarial and collaborative learning. In addition, simpler architectures reduce optimisation instability in adversarial settings and enable a fair like-for-like comparison across IL, AL, CL, and ACL. The architecture of the primary regressor includes an input layer, followed by a 50-unit dense layer, a 20-unit dense layer, and a final dense output layer, with the number of units in the output layer determined by the length of PH. The ReLU activation function is employed for all layers.

The auxiliary discriminator assesses the congruence between the actual sequences within the PH and those predicted by the primary regressor. This component processes a sequence that includes a specified length of genuine historical data, real or predicted PH data, and real post-PH data to determine whether the PH data is actual or predicted. A convolutional neural network (CNN), known for its classification capacity [72], was selected for the auxiliary discriminator. This network comprises an input layer, a 20-unit Conv1D layer, a 10-unit Conv1D layer, and a single-unit dense output layer with a sigmoid activation function, using binary cross-entropy as the loss function.

The auxiliary regressor plays a critical role in extending and validating the forecasting capacity of the primary regressor. It evaluates the predicted sequences by the primary regressor and uses them to project future trends beyond the immediate PH. The post-PH period is designed to be equal in length to the PH for consistency. By optimising collaborative loss at this stage, the auxiliary regressor component enables the primary regressor to return predictions that better predict future trends. To isolate the effect of the collaborative objective from the effect of changing model family, the auxiliary regressor was also implemented as an MLP with the same architecture as the primary regressor.

The primary regressor serves as the central component in all frameworks, while the auxiliary components—the discriminator and regressor—interconnect with the primary regressor to perform supplementary tasks. When paired with the auxiliary discriminator, the primary regressor engages in an adversarial interaction, generating sequences that not only optimise prediction accuracy but also challenge the discriminator’s ability to classify data correctly. In contrast, when linked with the auxiliary regressor, the primary regressor collaborates to produce sequences that minimise prediction error while enhancing the auxiliary regressor’s forecasting performance. Depending on whether the primary regressor operates independently or in conjunction with the auxiliary components, one independent and three interdependent frameworks are formed, as detailed below.

For clarity, the four frameworks can be summarised as follows. IL uses only the primary regressor and serves as the conventional baseline. AL connects the primary regressor to an auxiliary discriminator, introducing adversarial supervision within the prediction horizon. CL connects the primary regressor to an auxiliary regressor, introducing collaborative supervision through post-horizon forecasting. ACL combines both auxiliary components so that the primary regressor is trained under both adversarial and collaborative interactions.

### 3.1. Independent Learning

In the IL framework, the primary regressor operates alone, without the integration of any auxiliary components; similar to conventional BGP approaches, this method involves optimising a standard regression loss given in Equation (1).

(1) $${L_{S}=L}_{P}=E\left(PR\left(H_{x}\right),\;{PH}_{x}\right)$$

where $L_{S}$: system loss, $L_{P}$: predictive loss, E(a, b): error between a and b, ${PH}_{x}$: real prediction horizon sequence, $H_{x}$: real history sequence, *PR*: primary regressor, *PR*(*a*): primary regressor’s evaluation of *a*.

### 3.2. Adversarial Learning

**Figure 1:**
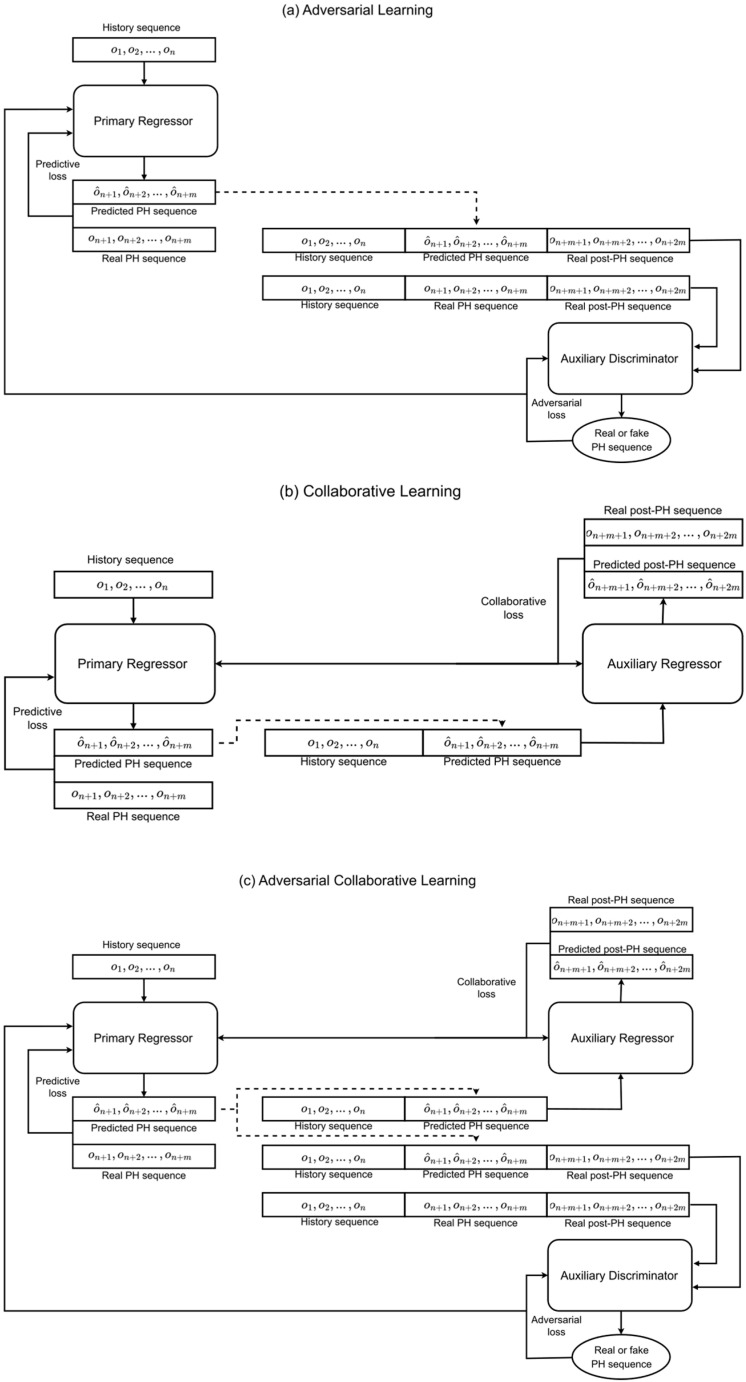
Data flow in the proposed learning frameworks. (**a**) In adversarial learning (AL), the real history sequence is input to the primary regressor (PR), which generates the predicted prediction-horizon (PH) sequence; the auxiliary discriminator (AD) then evaluates whether the PH segment is real or generated within the combined sequence context. (**b**) In collaborative learning (CL), the PR generates the PH prediction, which is then passed to the auxiliary regressor (AR) to forecast the post-PH sequence. (**c**) In adversarial collaborative learning (ACL), the PH prediction produced by the PR is evaluated simultaneously by the AD and the AR, introducing both adversarial and collaborative supervision during training. PR: primary regressor; AD: auxiliary discriminator; AR: auxiliary regressor. Note. n number of values in the history window; m: number of values in the prediction horizon and post-prediction-horizon interval; PH: prediction horizon; o: observation. Arrows indicate the direction of data flow between components during training.

As shown in Figure 1a, within the AL framework, the primary regressor is integrated with an auxiliary discriminator, and both components undergo simultaneous training. During training, the primary regressor interacts adversarially with the auxiliary discriminator. The loss functions in Equations (2) and (3) are optimised for the auxiliary discriminator and the primary regressor, respectively. These loss functions instruct the discriminator to classify real data as 0 and generated data as 1, while the primary regressor is trained to generate outputs that both enhance prediction accuracy and reduce the discriminator’s ability to classify generated data accurately. The system’s overall loss, as shown in Equation (4), is overall predictive and adversarial loss.

(2) $$L_{P}=E\left(PR\left(H_{x}\right),\;{PH}_{x}\right)$$

(3) $$L_{A}=E\left(AD\left({PH}_{x}\right),1\right)+E(AD({PH}_{\hat{x}}),0)$$

(4) $$L_{S}=L_{P}+L_{A}$$

where $L_{P}$: predictive loss, $L_{A}$: adversarial loss, $L_{S}$: system loss, *AD*: auxiliary discriminator, *E*(*a*, *b*): error between *a* and *b*, *AD*(*a*): auxiliary discriminator’s evaluation of *a*, ${PH}_{x}$: real prediction horizon sequence, ${PH}_{\hat{x}}$: synthesised prediction horizon sequence, $H_{x}$: real history sequence, *PR*: primary regressor, *PR*(*a*): primary regressor’s evaluation of *a*.

In practice, the adversarial framework in this study was trained using a fixed and consistent optimisation procedure across all scenarios. The primary regressor and auxiliary discriminator were updated jointly under the same epoch schedule, using ADAM optimiser. We intentionally adopted lightweight and stable subnetworks for this interaction, namely an MLP-based regressor and a CNN-based discriminator, in order to reduce optimisation volatility and maintain a balanced adversarial process. No additional adversarial stabilisation mechanisms, such as gradient penalties or specialised alternating update schedules, were introduced; rather, stability was pursued through architectural simplicity, consistent optimisation settings, and repeated-run evaluation.

### 3.3. Collaborative Learning

In the CL framework (Figure 1b), the primary regressor is connected to the auxiliary regressor. Both modules undergo simultaneous training, during which the primary regressor collaborates with the auxiliary regressor. The loss functions in Equations (5) and (6) are used for the auxiliary regressor and primary regressor, respectively. The primary regressor not only optimises its performance by minimising PH prediction errors but also improves the auxiliary regressor’s post-PH forecasting performance. The system optimises the predictive loss and collaborative loss as shown in Equation (7).

(5) $$L_{P}=E\left(PR\left(H_{x}\right),\;{PH}_{x}\right)$$

(6) $$L_{C}=E(AR\left(H_{x}+{PH}_{\hat{x}}\right),{PPH}_{x})$$

(7) $$L_{S}=L_{P}+L_{C}$$

where $L_{P}$: predictive loss, $L_{C}$: collaborative loss, $L_{S}$: system loss, E(a, b): error between a and b ${PH}_{x}$: real prediction horizon sequence, ${PH}_{\hat{x}}$: synthesised prediction horizon sequence, *AR*: auxiliary regressor, *AR*(*a*): auxiliary regressor’s evaluation of *a*, $H_{x}$: real history sequence, ${PPH}_{x}$: real post prediction horizon sequence, *PR*: primary regressor, *PR*(*a*): primary regressor’s evaluation of *a*.

### 3.4. Adversarial Collaborative Learning

The ACL framework (Figure 1c) integrates the primary regressor with both the auxiliary discriminator and auxiliary regressor. In this setting, all three modules are trained together, with the primary regressor engaging in both adversarial and collaborative interactions. The loss functions in Equations (8)–(11) are used for the auxiliary discriminator, auxiliary regressor, and primary regressor, respectively. The primary regressor learns to generate sequences that not only optimise prediction accuracy but also degrade the discriminator’s performance and improve the auxiliary regressor’s forecasting ability. The system’s overall loss function is the combination of the three losses as shown in Equation (11). In the present implementation, the ACL objective is formed as an unweighted combination of predictive, adversarial, and collaborative losses, as defined in Equation (11). No additional weighting coefficients were introduced to rebalance these three components. This choice was made deliberately to preserve a controlled comparison with IL, AL, and CL and to evaluate the effect of combining the three learning signals without framework-specific loss tuning.

(8) $$L_{P}=E\left(PR\left(H_{x}\right),\;{PH}_{x}\right)$$

(9) $$L_{A}=E\left(AD\left({PH}_{x}\right),1\right)+E(AD({PH}_{\hat{x}}),0)$$

(10) $$L_{C}=E(AR\left(H_{x}+{PH}_{\hat{x}}\right),{PPH}_{x})$$

(11) $$L_{S}=L_{P}+L_{A}+L_{C}$$

where $L_{P}$: predictive loss, $L_{A}$: adversarial loss, $L_{C}$: collaborative loss, $L_{S}$: system loss, AD: auxiliary discriminator, E(a, b): error between a and b, AD(a): auxiliary discriminator’s evaluation of a, ${PH}_{x}$: real prediction horizon sequence, ${PH}_{\hat{x}}$: synthesised prediction horizon sequence, *AR*: auxiliary regressor, *AR*(*a*): auxiliary regressor’s evaluation of *a*, $H_{x}$: real history sequence, ${PPH}_{x}$: real post prediction horizon sequence, *PR*: primary regressor, *PR*(*a*): primary regressor’s evaluation of a.

In ACL, all three components were trained under a unified optimisation schedule using the same optimiser and training hyperparameters across all experiments. This design was chosen to keep the comparison with IL, AL, and CL controlled and transparent. The objective was not to maximise adversarial sophistication through additional regularisation heuristics, but to examine whether the collaborative interaction can improve the learning dynamics and predictive utility of the primary regressor under a stable and consistent training setting.

## 4. Blood Glucose Prediction

To develop BGP systems, Ohio T1D datasets were utilised. The data were first preprocessed to meet the specific requirements of the problem space. Following this, the primary regressor was trained using the three proposed interdependent frameworks, alongside the conventional independent learning approach. The resulting systems underwent rigorous evaluation to assess their performance.

### 4.1. Dataset Description

For developing BGP systems, this work investigates two Ohio T1D datasets [73]. The Ohio T1D datasets are a reputable benchmark in the field of BGP, known for their comprehensiveness and real-world applicability, making it a robust and conclusive dataset for evaluating models in this domain [74,75,76,77,78,79]. Each dataset encompasses eight weeks’ worth of diabetes-related attributes for a cohort of six individuals with T1D [73]. The first dataset compiles data for four females and two males aged between 40 and 60 years [73]. It was released in 2018 for the first BGP challenge [73]. The second dataset comprises data for one female and five males within the age range of 20–80 years old [73]. This dataset was disseminated for the second BGP challenge in 2020 [73]. Hereafter, this paper refers to the former dataset as Ohio T1D 2018 and the latter as Ohio T1D 2020.

In line with prior univariate BGP studies, this work uses the CGM modality from the Ohio T1D datasets [80]. CGM values were collected every five minutes using Enlite continuous glucose monitoring sensors (Medtronic MiniMed, Northridge, CA, USA) [73]. Following the standard dataset protocol, the last 10 days of data for each individual were used as the testing set, while the preceding 46 days were used as the training set [73]. All models were trained exclusively on the training portion, and the testing portion remained completely unseen until evaluation. Table 1 summarises key statistical properties of the CGM data, while a fuller description is available in the original dataset documentation [73].

Although the Ohio T1D datasets are widely used benchmarks in BGP research, the cohort size remains relatively limited. Across the two dataset releases, the present study evaluates the proposed framework on a modest number of contributors, each with an individual-specific training and testing split. Consequently, the evaluation is well suited to assessing temporal generalisation within each person, because models are trained on earlier observations and tested on later unseen observations from the same individual. However, this design does not constitute a leave-one-patient-out or cross-patient generalisation study, and the findings should therefore be interpreted primarily as evidence of subject-specific predictive effectiveness on this benchmark.

### 4.2. Data Preprocessing

This subsection reports the preprocessing analysis operated on the Ohio T1D datasets before proceeding with the BGP modelling phase.

#### 4.2.1. Missing Value Imputation

In the first stage of the preprocessing, missing CGM values are handled. Linear interpolation is implemented to fill in missing values in the training set. However, missing values in the testing set are imputed utilising linear extrapolation. This technique avoids information leakage by ensuring that the systems do not use future information during evaluation. Accordingly, the resulting systems remain suitable for real-time prediction.

#### 4.2.2. Problem Reframing

The next preprocessing stage is translating the sequence-to-sequence BGP task to a supervised ML problem. For this purpose, a window with the length of history plus PH is rolled over the CGM series, creating a set of associated vectors. Each vector is then split into pairs of input and output sequences according to the length of history and PH. This operation renders a subset of associated input and output sequences necessary for supervised ML [81,82,83]. In this study, two prediction horizons (PHs) were investigated: 30 min and 60 min ahead. Considering the five-minute sampling frequency of CGM values in the Ohio T1D datasets, these correspond to forecasting 6 and 12 future timesteps, respectively. To exemplify, a rolling window with a length of 90 min forms a set of vectors for BGP 30 min in advance from 60 min of lag observations. Then, each vector is subdivided so that the first 60 min are used as the input sequence and the final 30 min as the associated output sequence. These two PHs were chosen to examine model behaviour under both a near-term clinically actionable setting and a more demanding longer-horizon forecasting setting.

### 4.3. Evaluation Analyses

The predictive performance of the developed systems is rigorously evaluated from both mathematical and clinical perspectives. Given the complex nature of BGP, a variety of evaluation metrics are essential for a comprehensive assessment, ensuring that both prediction accuracy and clinical relevance are thoroughly considered.

#### 4.3.1. Mathematical Evaluation

BGP errors are measured using three widely used regression metrics: root mean square error (*RMSE*), mean absolute error (*MAE*), and mean absolute percentage error (*MAPE*), given in Equations (12)–(14), respectively. Moreover, the coefficient of determination (*r*^2^), given in Equation (15), is used to quantify the agreement between the reference and predicted *BG* levels.

(12) $$RMSE=\sqrt{\frac{1}{N}(\sum_{n=1}^{N}({BG}_{n}-\hat{{BG}_{n}}{)}^{2}}$$

(13) $$MAE=\frac{1}{N}\sum_{n=1}^{N}\left|{BG}_{n}-\hat{{BG}_{n}}\right|$$

(14) $$MAPE=\frac{100}{N}\sum_{n=1}^{N}\left|\frac{{BG}_{n}-\hat{{BG}_{n}}}{{BG}_{n}}\right|$$

(15) $$r^{2}=1-\frac{\sum_{n=1}^{N}{\left({BG}_{n}-{B\hat{G}}_{n}\right)}^{2}}{\sum_{n=1}^{N}{\left({BG}_{n}-\bar{BG}\right)}^{2}}$$

where *N* denotes the number of samples in the testing set, $BG_{n}$ denotes the observed blood glucose value at the *n*-th sample, $\hat{{BG}_{n}}$ denotes the predicted blood glucose value at the n-th sample, and $\bar{BG}$ denotes the mean observed blood glucose value across the testing set.

#### 4.3.2. Clinical Evaluation

The following evaluation criteria were employed to assess the performance of the generated systems from the clinical point of view.

Surveillance Error (SE) is a metric developed to quantify the clinical risk associated with errors in BGP. It assigns a numerical value to each prediction, reflecting the potential clinical impact of any inaccuracies. The calculation of SE is comprehensive, taking into account the magnitude and direction of the prediction error, as well as its possible health implications. For a detailed breakdown of the SE calculation methodology, readers are directed to the thorough explanation provided in the original article cited as [84]. Succinctly, 0 < SE < 0.5 reflects no clinical risk, 0.5 < SE < 1.5 slight clinical risk, 1.5 < SE < 2.5 moderate clinical risk, 2.5 < SE < 3.5 high clinical risk, and 3.5 < SE extreme clinical risk. This work utilises the percentage of predictions with no clinical risk (SE < 0.5) and the average surveillance error (ASE) for predictions across the entire testing set as evaluation metrics.

The Matthews correlation coefficient (*MCC*) is a robust statistical measure employed to evaluate the quality of binary classifications. It excels in scenarios where data distribution across two classes is unbalanced, considering true and false positives and negatives to provide a balanced metric. This attribute makes *MCC* particularly valuable in delivering accurate insights into classification models, especially when precision and recall are pivotal [85]. In this work, *MCC* is calculated as Equation (16) to score the fulfilment of the systems’ predictions in correctly prognosticating the occurrence of adverse glycaemic events (BG < 70 mg/dL or >180 mg/dL) as opposed to euglycaemic events (70 mg/dL < BG < 180 mg/dL).

(16) $$MCC=\frac{TP\times TN-FP\times FN}{\sqrt{\left(TP+FP\right)\left(TP+FN\right)\left(TN+FP\right)\left(TN+FN\right)}}$$

where *TP* (true positive) represents the number of adverse glycaemic events foresaw truly by the BGP system, *TN* (true negative) represents the number of euglycaemic events foresaw truly, *FP* (false positive) represents the number of adverse glycaemic events foresaw falsely, and *FN* (false negative) represents the number of euglycaemic events foresaw falsely.

Taken together, these clinical metrics complement the mathematical error measures by indicating whether numerical improvements are likely to translate into safer and more useful predictions in practice. Lower SE and ASE values imply that prediction errors are less clinically hazardous, while a higher percentage of predictions with SE < 0.5 indicates that more forecasts fall within the no-risk region. Likewise, higher *MCC* values indicate improved ability to distinguish adverse glycaemic events from euglycaemic states, which is important for timely preventive action against hypo- and hyperglycaemia.

#### 4.3.3. Statistical Analysis

A notable limitation in many AI-driven BGP techniques is the lack of rigorous statistical evaluation, which is crucial for accurate model comparison. In BGP, unique challenges arise from the diversity of evaluation metrics and glycaemic variability across individuals, underscoring the need for robust statistical methods. This work addresses these challenges by implementing a comprehensive statistical analysis framework for BGP model comparison.

We first applied the non-parametric Friedman test [86] to globally assess model performance at a 5% significance level. The Friedman test was chosen for its suitability in comparing model outcomes across multiple scenarios without the assumption of normality [87], which is required in parametric tests like ANOVA [88]. The null hypothesis of the Friedman test posits that performance distributions across models are identical.

Upon detecting statistically significant differences in the global analysis, local pairwise comparisons were conducted using the Nemenyi test. To mitigate the risk of Type I errors in these multiple comparisons, we applied the Holm–Bonferroni correction. Finally, the results were visually represented using a ranking critical difference (CD) diagram, which illustrates statistically significant differences between models.

#### 4.3.4. Experimental Protocol

For each Ohio T1D dataset, experiments were conducted for all six individuals and for two prediction horizons (30 min and 60 min). In every scenario, four learning systems—IL, AL, CL, and ACL—were evaluated using the same training/testing split and the same preprocessing pipeline, so that performance differences could be attributed primarily to the learning framework. Each configuration was repeated ten times to account for stochastic variability, and results are reported as mean ± standard deviation across runs.

#### 4.3.5. Optimisation and Training Stability

All models were trained under a shared optimisation setting using the ADAM optimiser for 600 epochs, with batch size 128 and learning rate 0.002. The same training hyperparameters were used across all frameworks to avoid framework-specific tuning effects. These hyperparameters were selected as a common optimisation configuration and were intentionally kept fixed across all experiments. No framework-specific hyperparameter search was performed, because the objective of this study was to compare the learning strategies under controlled training conditions rather than to maximise the performance of each framework through separate tuning.

For AL and ACL, the interacting subnetworks were trained simultaneously under this common schedule. To support stable optimisation, the study employed lightweight and comparatively robust component models, namely MLP-based regressors and a CNN-based discriminator. Rather than relying on additional adversarial stabilisation techniques, stability was promoted through architectural simplicity, consistent optimisation settings, and repeated-run evaluation.

## 5. Results and Discussion

This section presents the outcomes of the evaluation analyses for generated BGP systems and the associated discussions. Because the developed algorithms are stochastic, each system was run ten times, and the results are reported as mean ± standard deviation (SD). In all training scenarios, the ADAM optimiser is utilised, with an epoch size of 600, a batch size of 128, and a learning rate of 0.002.

### 5.1. Evaluation Results

Table 2 and Table 3 display the evaluation results for systems created using the Ohio T1D 2018 and Ohio T1D 2020 datasets, respectively, under two prediction horizons: 30 min and 60 min ahead. Each table is compartmentalised with the results of 12 scenarios, i.e., BGP modelling for six data contributors in two PHs. For each scenario, four systems are created: one using the independent learning approach and three using the proposed interdependent learning frameworks.

Monochromatic colour coding is applied in the tables to visualise intra-scenario comparisons between learning platforms. To this end, the cells are shaded using four grey colours, where the darkest to lightest colour codes the outcomes for each metric from the first to the fourth rank.

**Figure 2:**
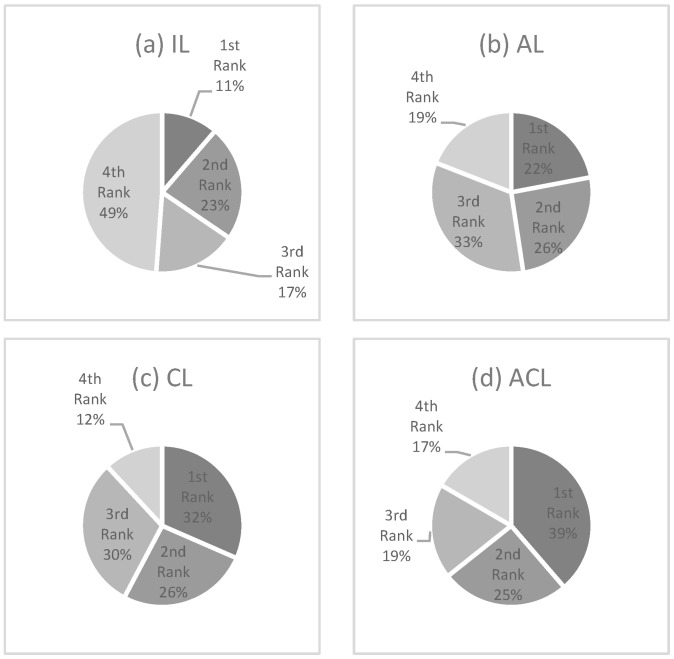
Summary of the evaluation metrics rankings obtained using each learning framework across all the scenarios considered for both studied datasets. Note. IL: independent learning; AL: adversarial learning; CL: collaborative learning; ACL: adversarial collaborative learning.

An inter-framework comparative analysis is performed, considering all evaluation metrics in all scenarios. To do so, Figure 2 graphically summarises the rankings for each learning framework as derived from the aggregated data in Table 2 and Table 3. The figure visually encapsulates the performance of each framework across all evaluated metrics and scenarios, thereby forming the empirical basis for the subsequent statistical scrutiny. ACL is shown to lead in performance, with the majority of its evaluations falling in the top two rankings, yielding an average rank of 2.14. This is followed by CL, AL, and IL, with average ranks of 2.22, 2.49, and 3.04, respectively.

**Figure 3:**
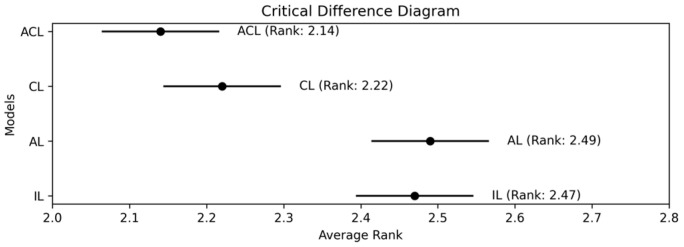
Results of critical difference analysis based on Nemenyi test for pairwise comparison of the developed models. Note. ACL: adversarial collaborative learning; CL: collaborative learning; AL: adversarial learning; IL: independent learning.

The results of the rank distribution, depicted in Figure 2, were further evaluated using the Friedman test, a non-parametric alternative to ANOVA that does not assume normality in the data. The Friedman test rejected the null hypothesis that all models have an identical distribution of ranks, with a *p*-value of 0.0265 at a significance level of 0.05. Consequently, we applied the Nemenyi post hoc test, complemented by critical CD analysis, as shown in Figure 3. In the CD diagram, the ranking of each model and the critical significance interval are displayed. Based on the results, the ACL model exhibited the best overall performance. The use of the collaborative unit in both CL and ACL models resulted in statistically significant improvements compared to models without the collaborative unit, specifically IL and AL.

Overall, the results emphasise the efficacy of the proposed collaborative component in advancing BGP compared to traditional adversarial and independent learning mechanisms. While adversarial interactions between the primary regressor and the discriminator ensure that the primary regressor produces sequences adhering to data distribution within the PH, this approach focuses solely on minimising adversarial loss. It does not consider how well the predictions reflect future trends beyond the PH. Specifically, the generated sequences are optimised to reduce dissimilarity with real CGM sequences within the PH but do not account for patterns predictive of future values.

In contrast, the collaborative relationship between the primary regressor and the auxiliary regressor adds an additional layer of evaluation to the primary regressor’s outputs. This interaction directs the primary regressor to produce sequences that incorporate greater knowledge of future trends. As a result, the trained primary regressor generates sequences that not only minimise prediction errors but also possess enhanced future predictivity. Through the dual interaction with both the adversarial and collaborative components, the primary regressor learns to generate sequences with optimal prediction errors, which are more consistent with the data dynamics within the PH and hold better predictive power for future trends.

Adversarial optimisation is known to be sensitive to instability; therefore, the present study intentionally adopted a restrained training design. Rather than introducing multiple adversarial regularisation heuristics, we used lightweight MLP and CNN subnetworks, a fixed optimisation procedure with ADAM, and the same hyperparameter settings across all frameworks. In AL and ACL, the interacting modules were trained simultaneously under this shared schedule. Furthermore, each experiment was repeated ten times and summarised as mean ± SD, which helps reveal whether performance is consistently reproducible despite stochastic optimisation. Within this controlled setting, the observed results indicate that the proposed frameworks can be trained in a stable and reliable manner for the Ohio T1D prediction tasks considered here.

Although recurrent and transformer-based architectures are often attractive for time-series modelling, this study intentionally adopts MLP-based regressors to isolate the contribution of the proposed learning strategy under a controlled architectural setting. Given the fixed-window univariate CGM formulation considered here, the MLP provides a computationally efficient and stable baseline while avoiding additional architectural confounding in the comparison among IL, AL, CL, and ACL. Moreover, the benchmark comparison in Table 4 shows that the resulting ACL framework remains competitive with an LSTM baseline. Nevertheless, more expressive temporal backbones may further strengthen the proposed framework and should be investigated in future work.

From a computational perspective, the proposed framework was intentionally designed to remain relatively lightweight. Although ACL is more demanding during training than IL, AL, or CL because it jointly optimises the primary regressor, auxiliary discriminator, and auxiliary regressor, the use of compact MLP- and CNN-based subnetworks helps keep this additional cost manageable. Importantly, the richer multi-component interaction is primarily a training-stage mechanism. At inference time, blood glucose forecasts are generated from fixed-length CGM input windows by the trained primary regressor, which supports efficient deployment. This distinction between a more involved training phase and a comparatively lightweight inference phase strengthens the practical feasibility of the proposed framework.

### 5.2. Benchmark Comparison with Established Baselines

To complement the controlled comparison among IL, AL, CL, and ACL, we also compare ACL against established blood glucose prediction baselines using the benchmark design reported in [89]. This benchmark includes a naive persistence baseline, classical statistical models, machine-learning models, and neural-network baselines, thereby positioning the proposed framework relative to widely used alternatives. This external comparison is intended to contextualise the proposed learning strategy beyond the within-study framework comparison.

Table 4 reports the performance of ACL alongside nine benchmark approaches on the Ohio T1D datasets, following the experimental design described in [89]. These baselines include a naive persistence model, classical statistical approaches, machine-learning methods, feed-forward neural networks, and an LSTM model. The comparison therefore provides an external reference point for assessing whether the proposed framework offers practical advantages beyond the within-study ablation analysis.

According to the benchmark results, ACL remains highly competitive against established BGP models and achieves the best overall average rank in the subsequent statistical comparison. To provide a more thorough evaluation, statistical analysis was conducted, similar to the approach described earlier. This involved ranking the models based on their average performance across all scenarios. Following this, a global statistical analysis was performed, using the Friedman test, to determine whether there was a significant difference in the performance of the models. The test decisively indicated the existence of statistically significant differences among the models.

**Figure 4:**
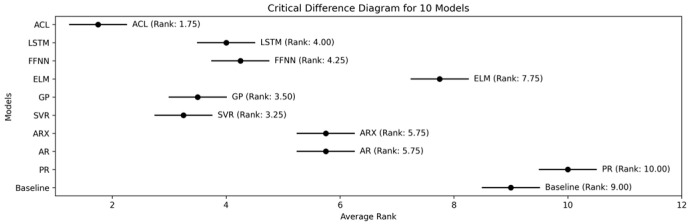
Results of critical difference analysis based on Nemenyi test for benchmark comparative analysis. Note. Baseline: a naive model that copies the last observation in the history window as future predictions. Abbreviations. PR: polynomial regression; AR and ARX: models from the ARIMAX family; SVR: support vector regression; GP: Gaussian process; ELM: extreme learning machines model; FFNN: feed-forward neural network model; LSTM: long short-term memory; ACL: adversarial collaborative learning.

Subsequently, a local pairwise statistical analysis using the Nemenyi test was conducted to further examine these differences. The outcome of the critical difference (CD) analysis is illustrated in Figure 4 where the rankings and significance intervals of each model are shown. From the diagram, it is evident that the ACL model outperforms all other models with an average rank of 1.75. Notably, the performance of ACL is statistically significantly better than that of the other models, as shown by its separation from the others in the CD diagram.

### 5.3. Clinical Impact

BGP represents a critical advancement in managing T1D, enabling more informed insulin dosing and other therapeutic interventions. The strong performance of the proposed ACL system in minimising prediction errors indicates its promise for improving the reliability of BGP on the Ohio T1D benchmark. Because the evaluation covers multiple individuals with differing glycaemic characteristics, the findings suggest that the framework is adaptable across varied subject profiles within this dataset. However, given the limited cohort size and the subject-specific evaluation design, these results should be interpreted cautiously and not as definitive evidence of broad population-level generalisability. Although the Ohio T1D datasets provide a recognised and clinically relevant benchmark, broader generalisation to external clinical cohorts remains to be established. Independent datasets may differ in patient demographics, glycaemic variability, treatment routines, sensor characteristics, and missingness patterns, all of which can influence predictive performance. In addition, real-world CGM streams may present further challenges such as sensor noise, temporary signal dropouts, calibration-related variability, and workflow-related latency. Therefore, the present findings should be interpreted as strong benchmark evidence, while validation on external clinical datasets and prospective real-world CGM streams remains an important next step.

When integrated with CGM systems, these predictive models provide real-time, personalised insights into glucose trends, supporting more precise and proactive diabetes management. Improved glycaemic control, reduced frequency of complications, and ultimately a higher quality of life for T1D patients could result from this approach. Furthermore, the adaptability of the proposed ACL system to individual patient data supports its role as an AI-enabled personalised medicine approach for subject-specific glucose forecasting and more tailored diabetes management. Incorporating these advanced models into clinical practice may enable healthcare providers to enhance the safety and efficacy of diabetes management, ultimately benefiting patient outcomes and potentially reducing the healthcare burden associated with T1D.

In a practical implementation, integration with CGM systems would involve continuously updating a rolling history window as new sensor measurements arrive and applying the trained primary regressor to generate the next 30 min or 60 min forecast. This workflow is compatible with real-time monitoring because the model operates on regularly sampled CGM inputs and does not require future information at inference. Nevertheless, translation into deployed CGM-supported decision systems would require prospective validation under live streaming conditions, where issues such as sensor interruptions, delayed readings, and user-specific behavioural variability may affect operational performance.

## 6. Conclusions

This work examined adversarial learning for blood glucose prediction, an area that remains underexplored despite the promise of adversarial approaches in other time-series forecasting domains. Traditional adversarial forecasting frameworks rely on a predictor-discriminator interaction to improve forecast realism within the target horizon. In contrast, the proposed method introduces an additional collaborative interaction through an auxiliary regressor that evaluates whether the predicted horizon remains useful for forecasting the immediately following post-horizon segment. The core novelty of ACL therefore lies in combining adversarial within-horizon supervision with collaborative post-horizon supervision in a single interdependent framework, rather than in merely extending a standard GAN-based predictor or adopting a conventional longer-horizon forecasting objective.

Experiments were conducted using two publicly available and well-known Ohio T1D datasets. The systems were rigorously evaluated through both mathematical regression metrics and clinically relevant performance measures. The results consistently demonstrated the effectiveness of the proposed architectures, showcasing their ability to handle the complexities of BGP. In addition to the controlled comparison among the four proposed learning frameworks, the study also benchmarked ACL against established blood glucose prediction models reported in the literature, including statistical approaches, machine-learning models, feed-forward neural networks, and an LSTM baseline. This broader comparison showed that ACL remains competitive with strong existing approaches while achieving the best overall average rank in the benchmark statistical analysis. These findings strengthen the evidence that the proposed learning strategy contributes meaningfully beyond the internal framework variants examined in this work.

In summary, the findings of this study underscore the potential of the enhanced adversarial learning frameworks to provide alternative learning mechanisms that surpass traditional approaches. Beyond methodological advancement, this work contributes to personalised medicine by supporting more individualized blood glucose forecasting from CGM data, with potential value for patient-specific monitoring, glycaemic risk management, and future AI-enabled diabetes decision support

## 7. Future Work and Potential Improvements

Several promising avenues remain for further development and improvement of this work, each addressing opportunities to expand on current limitations and deepen the impact of the proposed approach.

Effective data fusion in TSF is complex, particularly when incorporating diverse modalities from various sources. Adapting the proposed methods in this study to handle multivariate TSF is a logical next step. Future work could expand the proposed framework by introducing a conditional ACL system, where auxiliary variables are integrated as conditional inputs. This would allow for a comprehensive understanding of complex TS patterns where multiple influencing factors are present.

This work establishes a novel learning strategy specifically tailored for BGP. Given its potential, future studies should explore its application in other TSF domains where comparable volatility and complexity are present, such as energy demand forecasting, healthcare monitoring, or financial trends. In addition, while this study intentionally prioritised evaluation of the learning strategy under stable and lightweight architectures, future research could investigate replacing the MLP-based regressors with recurrent or transformer-based backbones to determine whether stronger temporal encoders further enhance the proposed ACL framework. Identifying model variants best suited for each domain could improve both the generalisability and performance of the approach.

A further consideration is the limited cohort size of the Ohio T1D datasets. While these datasets are established and relevant benchmarks, they include a relatively small number of individuals, and the present experimental design evaluates each model on held-out future data from the same individual rather than on entirely unseen patients. Accordingly, the reported findings provide strong evidence of within-patient temporal generalisation on a recognised benchmark, but broader clinical generalisation should be confirmed in future studies using larger and more diverse cohorts and dedicated cross-patient validation protocols.

## 8. Data and Code Availability

Instructions on attaining the Ohio T1D datasets can be found at this address. Also, we have made our source codes accessible on this Gitlab repository. For these implementations, we scripted in Python (3.6.7) [90]. The third-party libraries used include TensorFlow [91], Keras [92], Pandas [93], NumPy [94], Sklearn [95], and statsmodels [96]. Training was conducted on an Apple MacBook Pro () equipped with an Apple M1 Pro chip, 16 GB unified memory, and the integrated Apple M1 Pro GPU. Under this configuration, training a single ACL model for one subject-horizon scenario required approximately 20 min, while inference on a single input window was comparatively lightweight because deployment relies only on the trained primary regressor.

## Figures and Tables

**Table 1:** Demographic information of contributors and summary of statistical properties of blood glucose data (the focal modality) in the Ohio datasets.

Ohio Dataset	PID	Sex	Age	Set	Blood Glucose Data							
					Count	Range (mg/dL)	Mean (mg/dL)	SD (mg/dL)	MR (%)	HOR (%)	ER (%)	HRR (%)
2018	559	female	40–60	Training	10,655	40–400	167.53	70.44	12.06	3.65	55.98	40.37
				Testing	2444	45–400	168.93	67.78	14.81	3.03	59.86	37.11
	563	male	40–60	Training	11,013	40–400	146.94	50.51	8.80	2.82	72.81	24.36
				Testing	2569	62–313	167.38	46.15	4.71	0.70	60.45	38.85
	570	male	40–60	Training	10,981	46–377	187.5	62.33	5.73	1.97	42.97	55.07
				Testing	2672	60–388	215.71	66.99	5.05	0.41	29.04	70.55
	575	female	40–60	Training	11,865	40–400	141.77	60.27	10.43	8.71	68.62	22.66
				Testing	2589	40–342	150.49	60.53	4.94	5.37	63.50	31.13
	588	female	40–60	Training	12,639	40–400	164.99	50.51	3.69	1.04	63.56	35.40
				Testing	2606	66–354	175.98	48.66	3.42	0.15	53.26	46.58
	591	female	40–60	Training	10,846	40–397	156.01	58.03	17.59	3.94	63.97	32.09
				Testing	2759	43–291	144.83	51.42	3.15	5.18	67.27	27.55
2020	540	male	20–40	Training	11,914	40–369	136.78	54.75	9.76	7.08	72.66	20.25
				Testing	2360	52–400	149.94	66.46	6.74	5.64	68.18	26.19
	544	male	40–60	Training	10,533	48–400	165.12	60.08	19.11	1.47	63.78	34.75
				Testing	2715	62–335	156.48	54.14	15.47	1.22	68.29	30.50
	552	male	20–40	Training	8661	45–345	146.88	54.63	22.30	3.89	72.05	24.06
				Testing	1792	47–305	138.11	50.23	85.71	3.57	80.02	16.41
	567	female	20–40	Training	10,750	40–400	154.43	60.88	24.91	6.75	63.40	29.84
				Testing	2388	40–351	146.25	55.00	20.18	8.33	67.38	24.29
	584	male	40–60	Training	12,027	40–400	192.34	65.29	9.13	0.80	47.69	51.51
				Testing	2661	41–400	170.48	60.76	12.40	1.01	61.86	37.13
	596	male	60–80	Training	10,858	40–367	147.17	49.34	25.35	2.08	73.99	23.93
				Testing	2663	49–305	146.98	50.79	9.76	2.78	75.07	22.16

Note. PID: patient identification; SD: standard deviation; MR: missingness rate; HOR: hypoglycaemic rate; ER: euglycaemic rate; HRR: hyperglycaemic rate. Hypoglycaemia, euglycaemia, and hyperglycaemia refer to when the blood glucose level is, respectively, less than 70 mg/dL, between 70 and 180 mg/dL, and more than 180 mg/dL. Both hypoglycaemia and hyperglycaemia are adverse glycaemic events.

**Table 2:** Evaluation results for blood glucose prediction systems created using the Ohio T1D 2018 dataset.

Scenario	System	Evaluation Metric						
		RMSE ± SD (mg/dL)	MAE ± SD (mg/dL)	MAPE ± SD (%)	r^2^ ± SD (%)	MCC ± SD (%)	SE < 0.5 ± SD (%)	ASE ± SD
PID 559 PH 30	IL	19.36 ± 0.43	13.45 ± 0.22	8.79 ± 0.19	91.71 ± 1.20	81.08 ± 0.86	89.96 ± 0.56	0.189 ± 0.002
	AL	18.87 ± 0.32	13.29 ± 0.14	8.65 ± 0.12	92.12 ± 1.12	80.01 ± 0.98	90.29 ± 0.95	0.185 ± 0.003
	CL	19.05 ± 0.34	13.17 ± 0.18	8.78 ± 0.17	91.97 ± 1.01	80.68 ± 0.89	89.52 ± 0.51	0.184 ± 0.001
	ACL	18.77 ± 0.18	13.09 ± 0.12	8.55 ± 0.10	92.20 ± 0.92	81.37 ± 0.87	89.60 ± 0.62	0.184 ± 0.002
PID 559 PH 60	IL	33.22 ± 0.65	25.08 ± 0.40	17.97 ± 0.38	75.58 ± 0.96	64.43 ± 0.88	75.78 ± 0.92	0.350 ± 0.003
	AL	32.54 ± 0.94	23.88 ± 0.47	16.19 ± 0.41	76.57 ± 0.89	65.57 ± 0.74	78.67 ± 0.91	0.329 ± 0.005
	CL	32.07 ± 0.56	24.26 ± 0.32	16.00 ± 0.27	75.80 ± 0.75	65.27 ± 0.62	78.11 ± 0.62	0.327 ± 0.002
	ACL	32.54 ± 0.49	24.12 ± 0.24	16.55 ± 0.20	76.57 ± 0.67	64.71 ± 0.54	77.43 ± 0.42	0.336 ± 0.007
PID 563 PH 30	IL	19.68 ± 0.21	13.37 ± 0.19	8.23 ± 0.17	81.81 ± 0.67	74.17 ± 0.86	91.68 ± 1.01	0.183 ± 0.004
	AL	19.05 ± 0.18	14.02 ± 0.16	8.19 ± 0.15	80.42 ± 0.71	73.46 ± 0.51	91.64 ± 0.95	0.188 ± 0.002
	CL	19.17 ± 0.22	13.61 ± 0.16	8.13 ± 0.13	81.75 ± 0.72	73.60 ± 0.42	91.56 ± 0.62	0.187 ± 0.003
	ACL	18.75 ± 0.12	13.05 ± 0.10	8.15 ± 0.09	82.37 ± 0.51	77.10 ± 0.49	91.95 ± 0.79	0.182 ± 0.001
PID563 PH 60	IL	29.97 ± 0.84	21.87 ± 0.53	13.81 ± 0.42	57.81 ± 0.74	57.97 ± 0.28	81.23 ± 0.34	0.305 ± 0.006
	AL	30.57 ± 0.54	22.06 ± 0.32	13.65 ± 0.24	56.11 ± 0.62	56.61 ± 0.59	80.71 ± 0.47	0.301 ± 0.004
	CL	30.65 ± 0.62	21.99 ± 0.36	13.63 ± 0.21	55.88 ± 0.28	56.99 ± 0.39	80.52 ± 0.51	0.301 ± 0.001
	ACL	31.04 ± 0.41	22.64 ± 0.26	13.65 ± 0.14	54.73 ± 0.39	53.13 ± 0.62	79.50 ± 0.61	0.306 ± 0.006
PID 570 PH 30	IL	16.25 ± 0.35	11.41 ± 0.28	5.68 ± 0.14	94.05 ± 0.81	86.49 ± 0.56	96.44 ± 0.79	0.107 ± 0.002
	AL	16.08 ± 0.30	11.25 ± 0.12	5.73 ± 0.12	94.17 ± 0.45	86.80 ± 0.68	96.40 ± 1.25	0.109 ± 0.004
	CL	16.47 ± 0.28	11.21 ± 0.14	5.60 ± 0.11	93.88 ± 0.31	86.79 ± 0.98	96.62 ± 0.68	0.105 ± 0.003
	ACL	15.86 ± 0.38	11.07 ± 0.11	5.58 ± 0.09	94.18 ± 0.64	86.73 ± 0.57	96.58 ± 0.72	0.104 ± 0.002
PID 570 PH 60	IL	29.89 ± 0.61	22.26 ± 0.42	10.91 ± 0.20	79.86 ± 0.96	75.79 ± 0.95	89.89 ± 0.65	0.203 ± 0.003
	AL	28.38 ± 0.84	20.65 ± 0.24	10.31 ± 0.18	81.85 ± 0.92	76.76 ± 0.78	90.11 ± 0.91	0.193 ± 0.004
	CL	27.37 ± 0.65	19.94 ± 0.18	10.39 ± 0.16	83.10 ± 0.54	77.55 ± 0.69	90.26 ± 0.57	0.193 ± 0.003
	ACL	27.5 ± 0.23	20.05 ± 0.12	10.48 ± 0.08	82.95 ± 0.53	77.88 ± 0.62	90.41 ± 0.64	0.195 ± 0.002
PID 575 PH 30	IL	23.01 ± 0.44	14.34 ± 0.32	10.13 ± 0.12	85.59 ± 0.50	76.08 ± 0.87	86.37 ± 0.84	0.222 ± 0.004
	AL	23.00 ± 0.31	14.85 ± 0.21	10.54 ± 0.29	83.91 ± 0.74	77.23 ± 0.32	88.00 ± 0.69	0.224 ± 0.004
	CL	22.45 ± 0.61	15.11 ± 0.53	10.16 ± 0.16	86.28 ± 0.64	77.31 ± 0.57	86.75 ± 0.72	0.219 ± 0.0025
	ACL	22.52 ± 0.25	15.16 ± 0.21	10.03 ± 0.17	86.20 ± 0.81	77.81 ± 0.61	86.99 ± 0.56	0.217 ± 0.003
PID 575 PH 60	IL	36.82 ± 0.53	26.30 ± 0.32	18.90 ± 0.20	63.12 ± 0.69	52.24 ± 0.62	70.89 ± 0.85	0.396 ± 0.006
	AL	36.25 ± 0.62	25.97 ± 0.28	18.80 ± 0.24	66.26 ± 0.82	52.37 ± 0.54	71.80 ± 0.92	0.395 ± 0.007
	CL	35.15 ± 0.87	25.57 ± 0.42	19.38 ± 0.14	66.09 ± 0.86	54.11 ± 0.89	70.85 ± 0.59	0.398 ± 0.005
	ACL	36.55 ± 0.82	26.07 ± 0.29	19.07 ± 0.19	64.65 ± 0.58	52.02 ± 0.38	70.03 ± 0.67	0.397 ± 0.004
PID 588 PH 30	IL	19.25 ± 0.51	13.78 ± 0.32	8.29 ± 0.21	83.95 ± 0.87	71.09 ± 0.84	92.02 ± 0.69	0.183 ± 0.001
	AL	18.81 ± 0.39	13.67 ± 0.24	8.14 ± 0.22	84.67 ± 0.59	73.05 ± 0.89	91.91 ± 0.92	0.198 ± 0.002
	CL	18.41 ± 0.42	13.58 ± 0.26	8.16 ± 0.13	85.32 ± 0.74	75.24 ± 0.52	92.02 ± 0.51	0.184 ± 0.001
	ACL	18.72 ± 0.35	13.64 ± 0.14	8.25 ± 0.08	84.82 ± 0.92	76.24 ± 0.43	91.73 ± 0.68	0.182 ± 0.003
PID 588 PH 60	IL	31.04 ± 0.42	23.06 ± 0.26	14.18 ± 0.19	58.25 ± 0.62	59.40 ± 0.63	78.17 ± 0.54	0.309 ± 0.003
	AL	30.92 ± 0.18	22.63 ± 0.12	13.90 ± 0.16	56.56 ± 0.42	57.36 ± 0.51	80.05 ± 0.58	0.277 ± 0.003
	CL	31.19 ± 0.59	22.97 ± 0.23	13.62 ± 0.18	58.56 ± 0.20	58.36 ± 0.24	79.07 ± 0.92	0.303 ± 0.004
	ACL	31.75 ± 0.51	23.14 ± 0.27	13.56 ± 0.23	56.32 ± 0.54	56.01 ± 0.26	80.30 ± 0.67	0.289 ± 0.004
PID 591 PH 30	IL	21.13 ± 0.17	15.61 ± 0.14	11.95 ± 0.13	81.92 ± 0.54	62.66 ± 0.52	82.79 ± 0.74	0.264 ± 0.003
	AL	21.41 ± 0.25	15.24 ± 0.21	11.83 ± 0.15	82.46 ± 0.68	65.11 ± 0.46	82.61 ± 0.72	0.262 ± 0.002
	CL	21.22 ± 0.20	15.68 ± 0.15	11.63 ± 0.13	82.77 ± 0.41	63.59 ± 0.49	82.90 ± 0.46	0.259 ± 0.001
	ACL	21.25 ± 0.24	15.49 ± 0.16	12.13 ± 0.14	82.73 ± 0.86	62.97 ± 0.52	82.10 ± 0.61	0.268 ± 0.003
PID 591 PH 60	IL	33.48 ± 0.56	25.88 ± 0.28	21.08 ± 0.25	57.11 ± 0.42	44.65 ± 0.51	68.38 ± 0.88	0.418 ± 0.008
	AL	32.75 ± 0.84	25.15 ± 0.40	20.75 ± 0.24	58.19 ± 0.68	44.61 ± 0.62	69.12 ± 0.71	0.407 ± 0.006
	CL	33.20 ± 0.54	25.53 ± 0.27	19.73 ± 0.19	58.96 ± 0.77	45.69 ± 0.66	69.23 ± 0.75	0.418 ± 0.002
	ACL	33.00 ± 0.20	25.45 ± 0.16	20.08 ± 0.12	58.98 ± 0.20	46.52 ± 0.51	69.44 ± 0.35	0.413 ± 0.001

Note. Outcomes of each evaluation metric for a given modelling scenario are colour-coded from dark grey for best to light grey for worst outcomes. Abbreviations. T1D: type 1 diabetes; RMSE: root mean square error; MAE: mean absolute error; MAPE: mean absolute percentage error; r^2^: coefficient of determination; SE: surveillance error; ASE: average surveillance error; MCC: Matthews correlation coefficient; PID: patient identity; PH: prediction horizon; IL: independent learning; AL: adversarial learning; CL: collaborative learning; ACL: adversarial collaborative learning.

**Table 3:** Evaluation results for blood glucose prediction systems created using the Ohio T1D 2020 dataset.

Scenario	System	Evaluation Metric						
		RMSE ± SD (mg/dL)	MAE ± SD (mg/dL)	MAPE ± SD (%)	r^2^ ± SD (%)	MCC ± SD (%)	SE < 0.5 ± SD (%)	ASE ± SD
PID 540 PH 30	IL	21.49 ± 0.21	15.93 ± 0.16	11.81 ± 0.14	89.72 ± 0.68	73.84 ± 0.74	81.05 ± 0.84	0.228 ± 0.003
	AL	20.90 ± 0.12	15.63 ± 0.10	11.17 ± 0.08	90.01 ± 0.86	74.46 ± 0.76	82.54 ± 0.67	0.238 ± 0.004
	CL	21.68 ± 0.32	16.08 ± 0.15	11.02 ± 0.12	89.58 ± 0.95	74.70 ± 0.84	81.98 ± 0.93	0.231 ± 0.002
	ACL	21.18 ± 0.44	15.75 ± 0.24	11.07 ± 0.18	90.26 ± 0.62	73.98 ± 0.62	84.69 ± 0.74	0.234 ± 0.001
PID 540 PH 60	IL	40.10 ± 0.62	30.59 ± 0.81	21.71 ± 0.45	64.88 ± 0.54	53.71 ± 0.47	61.20 ± 0.56	0.424 ± 0.005
	AL	38.76 ± 0.54	29.77 ± 0.65	21.96 ± 0.42	65.54 ± 0.62	56.92 ± 0.58	62.85 ± 0.48	0.419 ± 0.006
	CL	39.01 ± 0.53	30.06 ± 0.62	21.20 ± 0.35	65.50 ± 0.42	54.88 ± 0.52	65.12 ± 0.69	0.416 ± 0.007
	ACL	38.84 ± 0.84	29.48 ± 0.68	20.67 ± 0.32	66.43 ± 0.61	57.62 ± 0.48	64.51 ± 0.38	0.404 ± 0.001
PID 544 PH 30	IL	18.02 ± 0.61	13.06 ± 0.36	8.94 ± 0.19	88.61 ± 0.69	79.21 ± 0.69	91.15 ± 1.08	0.192 ± 0.005
	AL	17.99 ± 0.28	12.70 ± 0.18	8.35 ± 0.12	88.74 ± 0.79	79.45 ± 0.62	91.60 ± 1.12	0.180 ± 0.001
	CL	17.98 ± 0.29	12.61 ± 0.17	8.23 ± 0.11	88.74 ± 0.86	79.67 ± 0.57	92.08 ± 0.85	0.175 ± 0.002
	ACL	18.01 ± 0.32	12.72 ± 0.16	8.21 ± 0.15	88.72 ± 0.92	79.69 ± 0.54	91.89 ± 0.60	0.174 ± 0.002
PID544 PH 60	IL	31.39 ± 0.59	23.65 ± 0.56	16.05 ± 0.46	65.71 ± 0.60	58.59 ± 0.74	74.03 ± 0.64	0.336 ± 0.005
	AL	30.93 ± 0.42	23.21 ± 0.35	15.83 ± 0.23	66.72 ± 0.55	60.77 ± 0.42	75.14 ± 0.56	0.328 ± 0.001
	CL	30.85 ± 0.62	23.25 ± 0.31	16.01 ± 0.22	66.88 ± 0.43	61.05 ± 0.65	75.07 ± 0.48	0.333 ± 0.001
	ACL	31.47 ± 0.24	23.92 ± 0.21	16.65 ± 0.15	65.54 ± 0.39	61.25 ± 0.32	74.70 ± 0.72	0.341 ± 0.002
PID 552 PH 30	IL	16.73 ± 0.34	12.50 ± 0.14	9.70 ± 0.10	89.71 ± 0.98	74.76 ± 0.67	89.40 ± 0.86	0.205 ± 0.001
	AL	16.78 ± 0.13	12.50 ± 0.08	9.51 ± 0.08	89.65 ± 0.86	74.98 ± 0.54	89.87 ± 0.81	0.202 ± 0.003
	CL	16.89 ± 0.21	12.66 ± 0.11	9.70 ± 0.09	89.52 ± 0.84	75.14 ± 0.84	89.40 ± 0.82	0.205 ± 0.002
	ACL	16.69 ± 0.18	12.25 ± 0.11	9.35 ± 0.10	89.76 ± 0.69	74.97 ± 0.46	90.25 ± 0.39	0.197 ± 0.003
PID 552 PH 60	IL	29.42 ± 0.84	21.82 ± 0.54	16.53 ± 0.45	68.22 ± 0.38	59.36 ± 0.42	75.85 ± 0.84	0.331 ± 0.006
	AL	30.45 ± 0.54	23.76 ± 0.42	19.72 ± 0.36	65.93 ± 0.46	59.38 ± 0.65	73.50 ± 0.85	0.369 ± 0.003
	CL	29.40 ± 0.62	22.24 ± 0.42	17.52 ± 0.32	68.18 ± 0.86	60.13 ± 0.42	75.64 ± 0.86	0.342 ± 0.001
	ACL	28.96 ± 0.49	22.18 ± 0.36	17.71 ± 0.22	69.02 ± 0.84	61.41 ± 0.38	75.55 ± 0.94	0.341 ± 0.002
PID 567 PH 30	IL	20.68 ± 0.42	14.74 ± 0.21	11.01 ± 0.15	85.76 ± 0.58	64.45 ± 0.48	82.42 ± 0.59	0.258 ± 0.003
	AL	21.11 ± 0.65	15.25 ± 0.20	11.45 ± 0.14	85.02 ± 0.75	62.95 ± 0.52	81.81 ± 0.68	0.268 ± 0.005
	CL	20.88 ± 0.35	14.98 ± 0.15	11.18 ± 0.11	85.34 ± 0.69	64.03 ± 0.68	82.24 ± 0.84	0.260 ± 0.002
	ACL	20.57 ± 0.21	14.38 ± 0.12	10.28 ± 0.10	85.78 ± 0.76	68.08 ± 0.63	85.15 ± 0.20	0.232 ± 0.002
PID 567 PH 60	IL	38.41 ± 0.48	29.59 ± 0.35	23.81 ± 0.16	50.62 ± 0.68	58.04 ± 0.54	61.18 ± 0.18	0.503 ± 0.004
	AL	35.99 ± 0.81	26.68 ± 0.54	19.89 ± 0.32	56.46 ± 0.41	57.99 ± 0.16	64.43 ± 0.58	0.462 ± 0.003
	CL	37.42 ± 0.74	28.78 ± 0.42	22.71 ± 0.33	52.77 ± 0.68	59.13 ± 0.28	62.91 ± 0.92	0.486 ± 0.002
	ACL	36.93 ± 0.59	27.72 ± 0.37	21.26 ± 0.25	54.09 ± 0.82	58.03 ± 0.38	66.04 ± 0.54	0.440 ± 0.002
PID 584 PH 30	IL	21.78 ± 0.19	15.83 ± 0.13	10.56 ± 0.10	87.18 ± 0.91	77.08 ± 0.68	88.02 ± 0.91	0.211 ± 0.001
	AL	22.35 ± 0.22	16.12 ± 0.11	11.20 ± 0.08	86.40 ± 0.57	77.28 ± 0.57	87.54 ± 0.21	0.229 ± 0.002
	CL	21.32 ± 0.32	15.24 ± 0.13	9.89 ± 0.09	87.52 ± 0.65	78.05 ± 0.86	89.24 ± 0.52	0.203 ± 0.003
	ACL	22.41 ± 0.26	16.44 ± 0.14	11.25 ± 0.08	86.26 ± 0.42	77.24 ± 0.91	87.54 ± 0.84	0.229 ± 0.000
PID 584 PH 60	IL	36.14 ± 0.96	26.53 ± 0.74	17.06 ± 0.65	64.14 ± 0.87	64.12 ± 0.54	74.74 ± 0.62	0.343 ± 0.008
	AL	36.37 ± 0.71	26.79 ± 0.62	17.41 ± 0.42	63.68 ± 0.54	63.21 ± 0.58	74.55 ± 0.61	0.340 ± 0.006
	CL	36.82 ± 0.65	27.65 ± 0.42	18.74 ± 0.35	62.78 ± 0.25	61.75 ± 0.68	72.39 ± 0.58	0.366 ± 0.005
	ACL	36.04 ± 0.58	26.55 ± 0.38	17.34 ± 0.32	64.34 ± 0.36	63.27 ± 0.35	74.85 ± 0.46	0.343 ± 0.004
PID 596 PH 30	IL	17.92 ± 0.29	12.75 ± 0.21	9.54 ± 0.16	87.83 ± 0.88	75.67 ± 1.06	88.81 ± 0.89	0.203 ± 0.002
	AL	17.68 ± 0.41	12.44 ± 0.18	9.28 ± 0.13	87.70 ± 0.75	74.33 ± 0.96	89.07 ± 0.92	0.209 ± 0.002
	CL	17.42 ± 0.18	12.11 ± 0.11	8.84 ± 0.10	88.04 ± 0.71	74.58 ± 0.52	89.40 ± 0.81	0.193 ± 0.001
	ACL	17.83 ± 0.13	12.35 ± 0.06	8.89 ± 0.05	87.48 ± 0.68	73.53 ± 0.38	89.26 ± 0.50	0.196 ± 0.002
PID 596 PH 60	IL	29.41 ± 0.59	21.33 ± 0.42	15.89 ± 0.31	68.77 ± 0.84	54.61 ± 0.68	78.63 ± 0.88	0.321 ± 0.003
	AL	29.04 ± 0.65	20.91 ± 0.43	15.48 ± 0.32	66.80 ± 0.51	58.69 ± 0.54	79.47 ± 0.96	0.313 ± 0.000
	CL	28.93 ± 0.71	20.87 ± 0.49	15.31 ± 0.36	67.05 ± 0.68	57.50 ± 0.49	79.14 ± 0.72	0.312 ± 0.001
	ACL	29.21 ± 0.48	20.99 ± 0.36	15.10 ± 0.22	66.41 ± 0.40	56.29 ± 0.68	79.44 ± 0.13	0.311 ± 0.004

Note. Outcomes of each evaluation metric for a given modelling scenario are colour-coded from dark grey for best to light grey for worst outcomes. Abbreviations. T1D: type 1 diabetes; RMSE: root mean square error; MAE: mean absolute error; MAPE: mean absolute percentage error; r^2^: coefficient of determination; SE: surveillance error; ASE: average surveillance error; MCC: Matthews correlation coefficient; PID: patient identity; PH: prediction horizon; IL: independent learning; AL: adversarial learning; CL: collaborative learning; ACL: adversarial collaborative learning.

**Table 4:** Results of comparative analysis of mean accuracy performance for blood glucose prediction over Ohio T1D dataset.

Model	PH 30 min		PH 60 min	
	RMSE (mg/dL)	MAPE (%)	RMSE (mg/dL)	MAPE (%)
Baseline	28.32	13.51	41.02	20.37
PR	57.26	31.09	57.27	31.04
AR	20.70	9.62	33.20	16.73
ARX	20.61	9.59	33.43	16.73
SVR	20.10	9.88	32.27	17.38
GP	20.01	9.16	31.97	16.92
ELM	25.38	11.56	35.14	16.91
FFNN	21.00	9.33	32.93	15.83
LSTM	20.46	9.24	32.88	16.8
ACL	19.38	9.31	32.81	16.04

Note. Baseline: a naive model that copies the last observation in the history window as future predictions. Abbreviations. PR: polynomial regression; AR and ARX: models from the ARIMAX family; SVR: support vector regression; GP: gaussian process; ELM: extreme learning machines model; FFNN: feed-forward neural network model; LSTM: long short-term memory; ACL: adversarial collaborative learning; RMSE: root mean square error; MAPE: mean absolute prediction error.

## Data Availability

No new data were created or analyzed in this study.
